# The Influence of Microorganisms on the Onset and Development of Colorectal Cancer in Humans: A Descriptive Cross-Reference Study

**DOI:** 10.3390/life15030468

**Published:** 2025-03-15

**Authors:** Dragan M. Nikolic, Stojan Latincic, Jelena Jevtovic, Drasko Gostiljac, Vesna Stojiljkovic, Snezana Jovanovic, Ivan Soldatovic

**Affiliations:** 1 Faculty of Medicine, University of Belgrade, Dr Subotica 9, 11000 Belgrade, Serbia; stojan.latincic@gmail.com (S.L.); doctor@med.bg.ac.rs (D.G.); soldatovic.ivan@gmail.com (I.S.); 2 Clinic for Endocrinology, Diabetes and Metabolic Diseases-Laboratory for Human Pancreatic Islets, Dr Subotica 13, 11000 Belgrade, Serbia; 3 University Clinical Centre of Serbia, 11000 Belgrade, Serbia; jelenajev89@gmail.com (J.J.); drsnezana.jovanovic@gmail.com (S.J.); 4 Institute of Digestive Diseases, Clinic of Surgery, Dr Kosta Todorovic 6, 11000 Belgrade, Serbia; 5 Clinic for Gastroenterology and Hepatology, Dr Kosta Todorovic 2, 11000 Belgrade, Serbia; 6 Department of Molecular Biology and Endocrinology, “Vinča” Institute of Nuclear Sciences, National Institute of the Republic of Serbia, University of Belgrade, 11000 Belgrade, Serbia; vesnas@vin.bg.ac.rs; 7 Department of Microbiology, University Clinical Centre of Serbia, Višegradska 26, 11000 Beograd, Serbia; 8 Institute of Medical Statistics and Informatics, 11000 Belgrade, Serbia

**Keywords:** human colorectal cancer, *Escherichia coli*, *Enterococcus* spp., *Klebsiella*, *Enterobacter*, *Streptococcus gordonii*, *Pseudomonas aeruginosa*

## Abstract

Background: The aim of this study is to determine which types of microorganisms influence the onset and development of colorectal cancer (CRC) in humans. Methods: In patients with CRC, three swabs were taken for microbiological analysis during surgical removal of the cancer: the first swab from the surface of the healthy intestinal mucosa, the second from the surface of the tumor, and the third from the middle of the tumor tissue. Results: In the healthy mucosa of the colon, the most prevalent microorganism was *Escherichia coli* at 70.5%, followed by *Enterococcus* spp. (47.7%) and *Klebsiella/Enterobacter* (20.5%). Microbiological analysis of the swabs from the surface of the tumor tissue showed that *E. coli* was the most prevalent at 72.7%, followed by *Enterococcus* spp. at 40.9%, *Klebsiella/Enterobacter* at 25%, and *Pseudomonas aeruginosa* at 20%. In the center of tumor tissue, *E. coli* was the most prevalent at 77.3%, followed by *Enterococcus* spp. at 47.7%, *Klebsiella* at 27%, and *Pseudomonas aeruginosa* at 18.2%. Conclusion: Certain types of bacteria can influence the emergence and development of cancer, while other types can suppress the development of tumor tissue. Microbiological analysis of human stool samples can prevent the development of CRC.

## 1. Introduction

According to the International Agency for Research on Cancer (IARC), colorectal cancer (CRC) is, after lung cancer, the second most common cause of cancer mortality worldwide, with an estimated incidence rate of 935,173 deaths (IRD), or 9.0 per 100,000 people, in 2020 [1]. Incidence rates for CRC increased by 1–2% annually in young adults (aged < 55 years). In the 1990s, CRC was the fourth-leading cause of cancer death in both men and women younger than 50 years, but it is now the first in men and the second in women [2]. In 2020, more than 1.9 million cases of colorectal cancer (CRC) were recorded, and almost 0.9 million patients died of CRC worldwide. There are several possible risk factors that may lead to the development of CRC: alcohol consumption, smoking, obesity, sedentary lifestyle, unhealthy diet (high intake of red and processed meat and fat) psychological stress, gender, genetic predisposition, family history of CRC, abdominopelvic radiation, personal history of other diseases, and intestinal microbiota [3]. The homeostatic microbiome (microbiota) is the set of all microorganisms that inhabit the human body and that have a key role in and influence on other physiological systems. Disruption of the homeostatic microbiome undoubtedly leads to the development of various diseases, as has been demonstrated for impaired insulin secretion and diabetes [4,5]. Decreased diversity of the gut microbiome, where one species suppresses another, is closely associated with the development of CRC [6]. Special analyses found a negative correlation between the Lachnospiraceae species in the gut and the risk of CRC, and a positive correlation between Porphyromonadaceae species, the genus Lachnospiraceae UCG010, the genus Lachnospiraceae, and the genus Selimonas in the gut and CRC risk. These findings suggest a causal relationship between the gut microbiome and the risk of CRC [7]. The aim of this study was to determine which types of microorganisms in patients with CRC may influence the development and progression of tumor tissue by analyzing the microbial composition of the mucosa on the surface of a healthy colon, the surface of tumor tissue, and in the center of tumor tissue.

## 2. Material and Methods

All patients with colorectal carcinoma were operated on at the Clinic for Digestive Surgery, UKCS, using the open method through medial laparotomy. Depending on the anatomical location of the tumor, and in accordance with oncological principles, right hemicolectomy and extended right hemicolectomy were performed with digestive tract reconstruction, creating a manual ileocolic termino-lateral anastomosis in two layers for carcinomas of the right colon and hepatic flexure. For carcinomas of the left colon and rectum, depending on the anatomical tumor location and disease stage, left hemicolectomy, upper rectal resection, and low rectal resection with stapled colorectal termino-terminal anastomosis without protection, as well as colo-anal termino-terminal anastomosis with protective ileostomy, were performed. From the time of diagnosis of CRC to the operation, one to two months passed at the most. Pathohistological findings indicated stages of CRC from 1 to 3. All subjects enrolled in this research provided informed consent, which was approved by the Ethics Committee on Human Research of UKCS, protocol number 420/25, and this protocol was deemed acceptable by the committee. From 2021, 44 patients were operated on, including 25 men and 19 women, aged between 26 and 87 years (61.11 ± 08). All patients were Caucasian. For research purposes, three swabs were taken: from the healthy mucosa of the colon, from the surface of the tumor tissue, and from the center of the tumor tissue after tumor resection. The patient material samples were cultured on blood agar plates, MacConkey agar, Salmonella–Shigella (SS) agar, Schaedler agar plates, Sabouraud dextrose agar, Schaedler broth, selenite broth, and thioglycolate broth. Solid media were inoculated semi-quantitatively (four-quadrant method) to estimate the number of colonies grown. The inoculated media were incubated under aerobic and anaerobic conditions at 33 °C ± 2 °C for 48 h. Enrichment media were subcultured. Identification was performed using standard microbiological methods and the VITEK2 Compact system (bioMérieux, France). Sensitivity testing of isolated strains was conducted using the Kirby–Bauer method (Mueller–Hinton II agar plates [Torlak, Belgrade, Serbia]) and the VITEK-Compact system. The results were interpreted based on EUCAST standards [8,9]. Due to the specific nature of the research and the percentage-based presentation of results, statistical tests were not conducted.

## 3. Results

**Figure 1:**
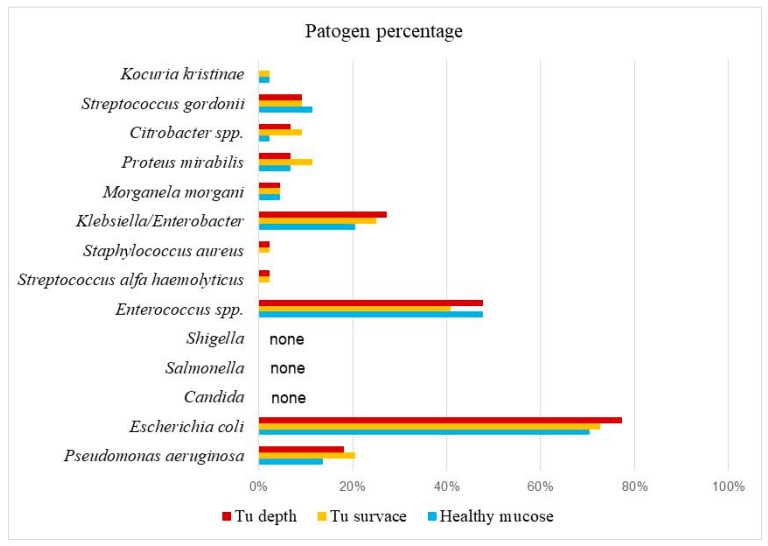
Graphic representation of the percentage of microorganisms in patients with colorectal cancer.

From 2021, 44 patients were operated on, including 25 men and 19 women, aged between 26 and 87 years (61.11 ± 08). In this study, microbiological analysis data were processed for 132 samples from 44 patients with CRC (colorectal carcinoma), and the results are presented in Table 1. A total of 15 microorganisms were identified. In the healthy mucosa of the colon, the most prevalent was *E. coli* at 70.5%, followed by *Enterococcus* spp. (47.7%) and *Klebsiella/Enterobacter* (20.5%). Other bacteria were found in smaller percentages: *Pseudomonas aeruginosa* 13.6%, *Proteus mirabilis* 6.8%, *Streptococcus gordonii* 11.4%, *Morganella morganii* 4.5%, *Citrobacter* spp., and *Kocuria kristinae* 2.3% (Table 1). Microbiological analysis of swabs from the surface of tumor tissue showed that *E. coli* was the most prevalent at 72.7%, followed by *Enterococcus* spp. at 40.9%, *Klebsiella/Enterobacter* at 25%, and *P. aeruginosa* at 20%. Other bacteria were present in percentages ranging from 2.3% to 11.4%. *Candida*, *Salmonella*, and *Shigella* were not detected (Table 1, Graph 1). In the center of tumor tissue, *E. coli* was the most prevalent in 77.3% of samples, followed by *Enterococcus* spp. in 47.7%, *Klebsiella* in 27%, and *P. aeruginosa* in 18.2%. Other bacteria were present in smaller amounts, ranging from 2.3% to 9.1% (Table 1). By comparing all three types of swabs, it is particularly interesting to note that *E. coli* appears in the highest percentage: 70.5% in healthy mucosa, 72.7% on the tumor surface, and 77.3% in the center of tumor tissue. *Enterococcus* spp. ranked second with 47.7%, 40.9%, and 47.7%, respectively, followed by *Klebsiella/Enterobacter* (20.5%, 25%, 27.3%) and *P. aeruginosa* (13.6%, 20.5%, 18.2%). *P. aeruginosa* was found in about 13% of samples from healthy mucosa, but its presence increased to up to 20% on the tumor surface and in tumor tissue (Table 1, Figure 1). *Candida*, *Salmonella*, and *Shigella* were not found in any of the three types of swabs (healthy mucosa surface, tumor surface, or tumor tissue center). Other bacteria were present in smaller percentages, ranging from 2.3% to 11.4%.

## 4. Discussion

The analysis of the results of this study identifies four types of bacteria, *E. coli*, *Enterococcus* spp., *Klebsiella/Enterobacter*, and *Streptococcus gordonii*, which are present (70–10%) in all three types of samples from the surface of the healthy intestinal mucosa, the surface of the tumor, and the core of the tumor tissue.

### 4.1. Escherichia coli

Among these bacteria, *E. coli* was the most prevalent on the surface of the healthy intestinal mucosa, the tumor surface, and within the tumor tissue, appearing in approximately 70% of cases. In addition to being one of the most common pathogens identified in medical oncology hospitals, *E. coli* is also a component of the saprophytic intestinal flora in humans and animals. Its presence is essential for digestion and the synthesis of certain substances, such as vitamin K. However, if the homeostasis of the microbiome is disrupted, an overgrowth of *E. coli* can lead to various diseases of the gastrointestinal tract, the urogenital system, and the lungs, and in severe cases, sepsis and meningitis. These conditions are most often caused by enteropathogenic, enterotoxic, and enteroinvasive types of *E. coli*. Community-acquired *E. coli* infections are generally uncommon and can be detected in asymptomatic cancer patients. Cancer has been shown to be a significant risk factor for contracting E. coli infections due to immune deficiencies. Neutropenic enterocolitis is a severe form of diarrhea that should be considered in cancer patients with diarrhea caused by *E. coli*. Persistent diarrhea in patients should prompt diagnostic evaluations for colorectal cancer (CRC), as such symptoms may indicate the presence of cancer in the intestinal system [10]. Recent literature reviews reveal that dysbiosis of the intestinal microbiota is a risk factor for CRC development, with polyketide synthase-positive *Escherichia coli* (pks+ *E. coli*) playing a key role in CRC pathogenesis [11]. Pks+ bacteria produce colibactin, a genotoxic protein that causes DNA damage in host colonocytes. Additionally, these bacteria promote genomic instability, disrupt the intestinal epithelial barrier, induce mucosal inflammation, modulate host immune responses, and influence cell cycle dynamics, creating a microenvironment conducive to tumor initiation and progression [12]. Biopsy samples analyzed in 1998 using PCR tools detected E. coli in 60% of adenomas and 77% of CRC cases, compared to 12% in adjacent normal biopsies and 3% in normal control samples [13]. Current studies have shown that the intestinal microbiota, including genera such as *Clostridium*, *Bacteroides*, *Enterococcus*, and *Escherichia*, can facilitate colorectal carcinogenesis through direct interaction with host cancer cells, generation of carcinogenic microbial metabolites, and secretion of oncogenic virulence factors [13,14,15,16].

### 4.2. Enterococcus *spp*.

Approximately 40% of the patients analyzed had *Enterococcus* bacteria on the surface of the healthy intestinal mucosa, the tumor surface, and in the core of tumor tissue (Table 1). Enterococci are part of the normal intestinal flora in humans and animals. The most well-known representatives of enterococci are *Enterococcus faecalis* and *Enterococcus faecium*, which can cause conditions such as endocarditis, urethritis, and sepsis. The connection between *Enterococcus* and colorectal cancer is evidenced by findings that co-incubation with conditioned medium of *E. faecalis* increased the proliferation of cultured CRC cells. Biliverdin (BV) was identified as a key metabolite produced by *E. faecalis*. BV promoted colony formation and cell proliferation while inhibiting cell cycle arrest in cultured CRC cells. BV also significantly increased the expression levels of IL-8 and VEGFA, accelerating angiogenesis in CRC [17]. Recent studies have shown that the use of auto-probiotics based on non-pathogenic strains of *Enterococcus faecium* and *Enterococcus hirae* as personalized functional food products (PFFPs) in the complex therapy of early-stage CRC reduced dyspeptic symptoms, postoperative complications, and serum levels of proinflammatory cytokines (IL-6 and IL-18) [18].

### 4.3. Klebsiella/Enterobacter

In this study, approximately 20% of the patients had this bacterium present in all three samples (on the surface of the healthy intestinal mucosa, on the tumor surface, and within tumor tissue). *Klebsiella* can be found in water, soil, plants, and insects, and is part of the normal flora of humans and animals, colonizing the nose, mouth, and intestines. *Klebsiella* species are opportunistic pathogens that can cause pneumonia, urinary tract infections, sepsis, meningitis, diarrhea, peritonitis, and soft tissue infections [19,20]. *Klebsiella/Enterobacter* is among the first intestinal colonizers in newborns. It was established that isolates of *Klebsiella pneumonia* (51-5) from the intestine of premature children can induce increased expression of proinflammatory genes with intestinal inflammation in ApcMin/+; Il 10−/− mice. Gnotobiotic experiments in Il10−/− mice have shown that a neonatal isolate induces intestinal inflammation in vivo, with increased expression of proinflammatory genes. Regulation of microbiota composition revealed that *K. pneumoniae* 51-5 accelerates the onset of inflammation in Il10−/− mice. Studies have shown that isolates of *K. pneumoniae* can induce intestinal inflammation and DNA damage, promoting tumor genesis [21].

### 4.4. Streptococcus gordonii

*Streptococcus gordonii* was detected in approximately 10% of patients across all three sample types. *S. gordonii* is a Gram-positive bacterium commonly found in the oral cavity as part of the saprophytic flora. *S. gordonii*, together with related organisms, constitutes a high percentage, up to 70%, of the bacterial biofilm that forms on clean tooth surfaces. As part of the saprophytic flora, it is harmless in the mouth; however, S. gordonii can cause acute bacterial endocarditis when systemically acquired [22]. Regarding the connection between CRC and the mentioned bacteria *S. gordonii*, there is one case reported in the literature of a patient with endocarditis and an advanced stage of CRC [23]. In our patients, no other systemic diseases were observed, but it is certain that S. gordoni has an influence on the development of CRC. It has been reported in the literature that *S. gordonii*, which participates in polybacterial infection of the gingiva causing periodontitis, also causes an increase in miRNA. Certain types of miRNA have also been reported in various forms of systemic diseases such as atherosclerosis, osteoarthritis, diabetes, obesity, and several cancers [24].

### 4.5. Pseudomonas aeruginosa

About 13% of *Pseudomonas* is found in the intestinal mucosa; however, its presence increases on the surface of the tumor and in the tumor tissue by up to 20%. *Pseudomonas aeruginosa* is a clinical bacterium that favors moist environments and is often the cause of various systemic infections in hospitalized patients who are under long-term antibiotic or immunosuppressive therapy. There are data showing that *P. aeruginosa* in human melanoma cell culture causes apoptosis of cancer cells [25]. *P. aeruginosa* produces one type of protein, Azurin, which negatively affects the growth and development of cancer cells in both human melanoma and breast cancer cells [26,27]. The very complex relationship between the aforementioned bacteria and cancer cells is shown by findings that cancer cells secrete aldolase, which increases the adhesion of *Bacillus pseudomonas* to the surface of cancer cells [28]. Examining the routes and types of infection in patients with adenocarcinoma of the pancreas, the presence of *P. aeruginosa* in the tumor tissue was determined in 33% of the subjects [29]. The fact that *P. aeruginosa* in pancreatic islet culture significantly reduces insulin secretion shows a very complex relationship between the mentioned bacteria and certain types of human host cells [30]. In addition to the fact that *Pseudomonas* has a very harmful effect and is a frequent cause of pneumonia and sinusitis in immunocompromised patients as well as complications in puncture wounds and burns, its presence in certain types of cancer obviously has a suppressive effect on the growth of cancer cells. So far, no data have been presented in the literature that indicate the existence and association of *P. aeruginosa* and colorectal cancer cells, so we can only assume that the presence of *Pseudomonas* can be favorable for CRC patients and negatively affect the development of CRC cells.

The participation of a larger number of patients was planned for this research. During the implementation of the research procedure, the COVID-19 pandemic occurred, so we were forced to suspend the cooperation between the clinics and the standard microbiological analysis of swabs.

The relationship between the gut microbiota and human health is very complex. Currently, approximately 1000 to 1150 species of bacteria have been identified in the human gut, with a core microbiota shared among different individuals consisting of approximately 160 species [31]. All patients who participated in the experiment stated that they were not undergoing long-term antibiotic therapy, which could affect the development of certain resistant strains of bacteria in the gastrointestinal tract. The patients received antibiotics (first-generation cephalosporins) immediately before the operation, which could not affect the microbiological findings. Please note that short-term antibiotic therapy lasting seven days, which patients may have taken in the previous period due to various infections, cannot permanently lead to a disruption of the homeostatic microbiome, because after the therapy is ceased, the previous state is very quickly restored, that is, the homeostatic microbiome returns to normal [32].

## 5. Conclusions

There is a very complex relationship between the microorganisms of the homeostatic microbiota in a healthy person, and their disruption due to the action of various factors can lead to the development of conditional pathogens and the development of various diseases, in this case CRC. Certain bacteria (*Escherichia coli*, *Enterococcus* spp., *Klebsiella*/*Enterobacter* and *Streptococcus gordonii*) favor the occurrence and development of CRC, while some (*Pseudomonas aeruginosa*) can suppress the growth of cancer cells. Regular analyses of the microbiological composition of the human stool can prevent the occurrence of CRC and it can be used as a referral for more sensitive diagnostic methods. Due to the competitive relationship of microorganisms of the saprophytic intestinal flora, in the future, we should investigate how to suppress the growth and overgrowth of pathogens that are associated with the occurrence and development of CRC.

## Figures and Tables

**Table 1:** Percentage presence of microorganisms in relation to the total number of patients with colorectal cancer, on the surface of the healthy intestinal mucosa, on the surface of the tumor, and in the middle of the tumor tissue.

	Group					
	Healthy Mucosa		Tumor Surface		Tumor Depth	
	N	%	N	%	N	%
*Pseudomonas aeruginosa*	6	13.6%	9	20.5%	8	18.2%
*Escherichia coli*	31	70.5%	32	72.7%	34	77.3%
*Candida*	0	0.0%	0	0.0%	0	0.0%
*Salmonella*	0	0.0%	0	0.0%	0	0.0%
*Shigella*	0	0.0%	0	0.0%	0	0.0%
*Enterococcus* spp.	21	47.7%	18	40.9%	21	47.7%
*Streptococcus alfa haemolyticus*	0	0.0%	1	2.3%	1	2.3%
*Staphylococcus aureus*	0	0.0%	1	2.3%	1	2.3%
*Klebsiella/Enterobacter*	9	20.5%	11	25.0%	12	27.3%
*Morganela morgani*	2	4.5%	2	4.5%	2	4.5%
*Proteus mirabilis*	3	6.8%	5	11.4%	3	6.8%
*Citrobacter* spp.	1	2.3%	4	9.1%	3	6.8%
*Streptococcus gordonii*	5	11.4%	4	9.1%	4	9.1%
*Kocuria kristinae*	1	2.3%	1	2.3%	0	0.0%

## Data Availability

Data are contained within the article.
